# Co-infection with trichomonas vaginalis increases the risk of cervical intraepithelial neoplasia grade 2–3 among HPV16 positive female: a large population-based study

**DOI:** 10.1186/s12879-020-05349-0

**Published:** 2020-09-01

**Authors:** Mei Yang, Lin Li, Chunfan Jiang, Xiaomin Qin, Min Zhou, Xiaogang Mao, Hui Xing

**Affiliations:** 1 Department of Obstetrics and Gynecology, Xiangyang Central Hospital, Affiliated Hospital of Hubei University of Arts and Science, Xiangyang, 441021 Hubei China; 2 Department of Pathology, Xiangyang Central Hospital, Affiliated Hospital of Hubei University of Arts and Science, Xiangyang, 441021 Hubei China

**Keywords:** Invasive cervical cancer, Cervical intraepithelial neoplasia, Human papillomavirus, Vaginal microenvironment, Trichomonas vaginitis

## Abstract

**Background:**

Evidence suggested that vaginal microbiome played a functional role in the progression of cervical lesions in female infected by HPV. This study aimed at evaluating the influence of common vaginal infection on the carcinogenicity of high risk HPV (hr-HPV).

**Methods:**

From January 15, 2017 to December 31, 2017, 310,545 female aged at least 30 years old had been recruited for cervical cancer screening from 9 clinical research centers in Central China. All the recruited participants received hr-HPV genotyping for cervical cancer screening and vaginal microenvironment test by a high vaginal swab. Colposcopy-directed biopsy was recommended for female who were infected with HPV 16 and HPV 18, and other positive hr-HPV types through test had undertaken triage using liquid-based cytology, cases with the results ≥ ASCUS among them were referred to colposcopy directly, and cervical tissues were taken for pathology examination to make clear the presence or absence of other cervical lesions.

**Results:**

Among 310,545 female, 6067 (1.95%) were tested with positive HPV 16 and HPV 18, 18,297 (5.89%) were tested with other positive hr-HPV genotypes, cervical intraepithelial neoplasia (CIN) 1, CIN 2, CIN 3 and invasive cervical cancer (ICC) were detected in 861 cases, 377 cases, 423 cases, and 77 cases, respectively. *Candida albicans* and Gardnerella were not associated with the detection of cervical lesions. Positive trichomonas vaginitis (TV) was correlated with hr-HPV infection (*p* < 0.0001). Co-infection with TV increased the risk of CIN 1 among female infected with hr-HPV (OR 1.18, 95% CI: 1.42–2.31). Co-infection with TV increased the risk of CIN 2–3 among female infected with HPV 16 (OR 1.71, 95% CI: 1.16–2.53).

**Conclusions:**

Co-infection of TV and HPV 16 is a significant factor for the detection of cervical lesions.

## Background

Cervical cancer is a common malignant tumor of the female reproductive system, and it progresses rapidly with a high mortality rate [1]. HPV is a DNA virus, which is widely found in nature. Hr-HPV (e.g. HPV 16, 18, 31, 35, 39, 45, 51, 52, 56, 58, 59, 68, 73, 82) among them are associated with high grade cervical intraepithelial neoplasia and invasive cervical cancer.

Under normal conditions, vaginal flora is composed of more than 20 kinds of microbes [2, 3]. Infection of the female reproductive tract refers to a series of infectious inflammation caused by the destruction of the defense system by microorganisms, such as viruses and bacteria [4]. The genital tract infection leads to an imbalance of the vaginal flora, which inhibits or reduces the lactobacillus substantially, which may cause the decline of the clearance of hr- HPV. Candida spp., Gardnerella and TV are the most common vaginal infections. As a part of the human commensal flora, Candida spp. always causes systemic and superficial infections [5]. Gardnerella vaginalis is considered as playing a vital role in the pathogenesis of bacterial vaginitis (BV) [6], and BV shows a correlation with severity of cervical neoplasia in HPV-positive female [7]. Still the aetiology and pathogenesis of BV are more complex, Gardnerella vaginalis is thought as a potential founder organism. As a sexually transmitted infectious agent, TV is found to cause local inflammation, and it affects the clearance of hr-HPV and contributes to cervical lesion detection in several research, but it is controversial [8]. Some studies have demonstrated a correlation between vaginal infection and the carcinogenicity of hr-HPV, but most of them are small-scale researches, and their conclusions are inconsistent [5, 9–16]. This research is with the most massive scale and the most comprehensive investigation about the correlation between cervical hr-HPV infection, CIN/ICC, and the vaginal microbiome.

Central China is one of the regions with the highest incidence of cervical cancer [17], and the social-economic conditions vary significantly in different districts. To study ICC influenced by multiple socioeconomic factors, nine areas in Central China with significantly different economic levels were included in this study. This study aimed at evaluating the influence of common vaginal infection on the carcinogenicity of hr-HPV.

## Methods

This project had been conducted in Central China from January 15, 2017 to December 31, 2017. Female aged at least 30 years old were recruited to undergo cervical cancer screening from 9 clinical research centers (Xiangzhou, Fancheng, Xiangcheng, Baokang, Nanzhang, Zaoyang, Yicheng, Gucheng and Laohekou) in Central China via media promotion and government notices. Female who received hysterectomy, were pregnant, without sexual history had been excluded. All the female had not been vaccinated against cervical cancer yet. All the participants received questionnaires, hr-HPV genotyping, and vaginal microbiota examination. The screening process was based on the interim clinical guidance of ASCCP in 2015 [18], female tested positive for HPV 16 and HPV 18 had been referred to colposcopy directly; female with other positive hr-HPV types through test had undertaken triage using liquid-based cytology, cases with the results ≥ ASCUS among them were referred to colposcopy directly. The protocol was approved by the Ethics Committee of Xiangyang Central Hospital.

### Questionnaires

All the recruited participants were interviewed by a trained interviewer and filled out a questionnaire (Additional file 1) for the first time. The content of the questionnaire includes age, marital status, ethnicity, the highest level of education, whether in menopause, date of last menstruation, history of past HPV infection, family history of cancer, reproductive history (number of pregnancies and number of births), method of contraception (contraceptive, condom and intrauterine contraceptive device), number of lifetime sex partners and whether in poverty (whether being a poverty alleviation target).

### HPV genotyping

All the recruited female underwent a gynecological examination. A cervical specimen was taken using a cervical brush, and a high vaginal swab was collected. COBAS4800 (Roche Molecular Systems, Alameda, CA) assay was used for typing HPV DNA. The COBAS 4800 HPV test detected a total of 14 h-HPV types simultaneously: HPV-16 individually, HPV-18 individually, and pooled hr-HPV genotypes other than HPV 16 and 18 (31, 33, 35, 39, 45, 51, 52, 56, 58, 59, 66 and 68), in addition to a separate high b-globin control.

### Vaginal microenvironment test

The high vaginal swab was processed for microscopic evaluation of vaginal microenvironment, including the presence of *Candida albicans*, TV, Gardnerella, clue cells, and miscellaneous bacteria (vaginal bacteria except for lactobacilli) density [19]. A vaginal wet mount was prepared for the detection of *Candida albicans*. Candida was diagnosed using the KOH method (10% KOH), and vaginal trichomonas was examined according to conventional methods, the swimming trichomonas was observed under the microscope (× 400). Gardnerella, clue cells, and miscellaneous bacteria density (MBD) were observed by Gram staining. The scoring method of miscellaneous bacteria density was listed as follows, and no miscellaneous bacteria was scored as I, 1–5 miscellaneous bacteria/OML as II, 6–30 miscellaneous bacteria/OML as III, >30 miscellaneous bacteria/OML as IV [19].

### Thinprep cytologic test

According to the manufacturer’s instructions, slides for liquid-based cytology were prepared. Bethesda System 2001 terminology was used for reporting the results [20]. The following five different categories were reported: negative for intraepithelial lesion or malignancy (NILM); atypical glandular cells (AGC); squamous intraepithelial lesion (SIL): low grade squamous intraepithelial lesion (LSIL) or high grade squamous intraepithelial lesion (HSIL); atypical squamous cells of undetermined significance (ASC-US) or atypical squamous cells not possible exclude HSIL (ASC-H) and squamous cell carcinoma (SCC).

### Colposcopy-directed biopsy and verification of disease status

Female with positive HPV 16 and HPV 18 through the test or with other hr-HPV types through the test and the result of liquid-based cytology ≥ ASCUS underwent further cervical biopsy and/or endocervical curettage (ECC) by a local trained gynecologist in each hospital. The cervical tissues underwent the preparation of histological sections. Two pathologists made the diagnosis separately. The histological diagnoses of cervical lesions were divided into normal, CIN1, CIN2, CIN3 and ICC according to the WHO criteria for the pathological staging of cervical lesions [21, 22]. CIN1(cervical intraepithelial neoplasia grade 1): cells showed light atypia and irregular arrangement, but remained polar, and the abnormal proliferated cells were limited to the subcortical 1/3. CIN2(cervical intraepithelial neoplasia grade 2): cells showed obvious atypia and disordered arrangement. Abnormal proliferated cells occupied 2/3 of the subcortical area. CIN3(cervical intraepithelial neoplasia grade 3): Severe atypical hyperplasia of the epithelial cells were significantly atypia, loss of polarity, abnormal proliferation of cells expanded to 2/3 of the epithelium or almost the entire layer, difficult to distinguish from carcinoma in situ. The epithelial atypia cells of carcinoma in situ involved the whole layer, with no polarity, significant nuclear atypia and more mitotic phases. The basement membrane of the epithelium was intact without stroma infiltration. ICC (invasive cervical cancer): Cancer cells break through the basement membrane. (I suggest describing in more detail the method used for pathological staging or including the reference that supports it.)

### Statistical analysis

Histological results (normal, CIN1, CIN2, CIN3 and ICC) were distributed among respective hr-HPV types and then evaluated. Univariate analysis was used for comparing whether the risk of any cervical lesions (tri categorical: CIN1, CIN2–3 and ICC) differed when female having a vaginal infection (*Candida albicans*, Gardnerella and TV) or not. Odds ratios and 95% confidence intervals (CIs) were calculated to study the specific risk among different HPV types. Next, social-demographic and reproductive characteristics were taken into consideration so as to improve the accuracy of the results. These characteristics of the participants were presented as proportions. Correlations between specific vaginal infection and hr-HPV genotype and CIN/ICC were assessed using stepwise logistic regression after adjusting for all potential risk factors. ORs and 95% CIs were calculated to analyze the correlation between possible risk factors and pathogenic infection. Data was analyzed with SPSS Software Version 20.0.

## Results

### The overall prevalence of hr-HPV, *Candida albicans*, Gardnerella and TV

Among 310,545 participants, 24,364 (7.84%) female were infected with hr-HPV. Candida albicans were detected.

In 13,763 (4.43%) female, Gardnerella were detected in 1050 (0.34%) female, and TV were tested positive in 5683 (1.83%) female, respectively.

### Distribution of HPV types in CIN and ICC female from Central China

**Fig. 1:**
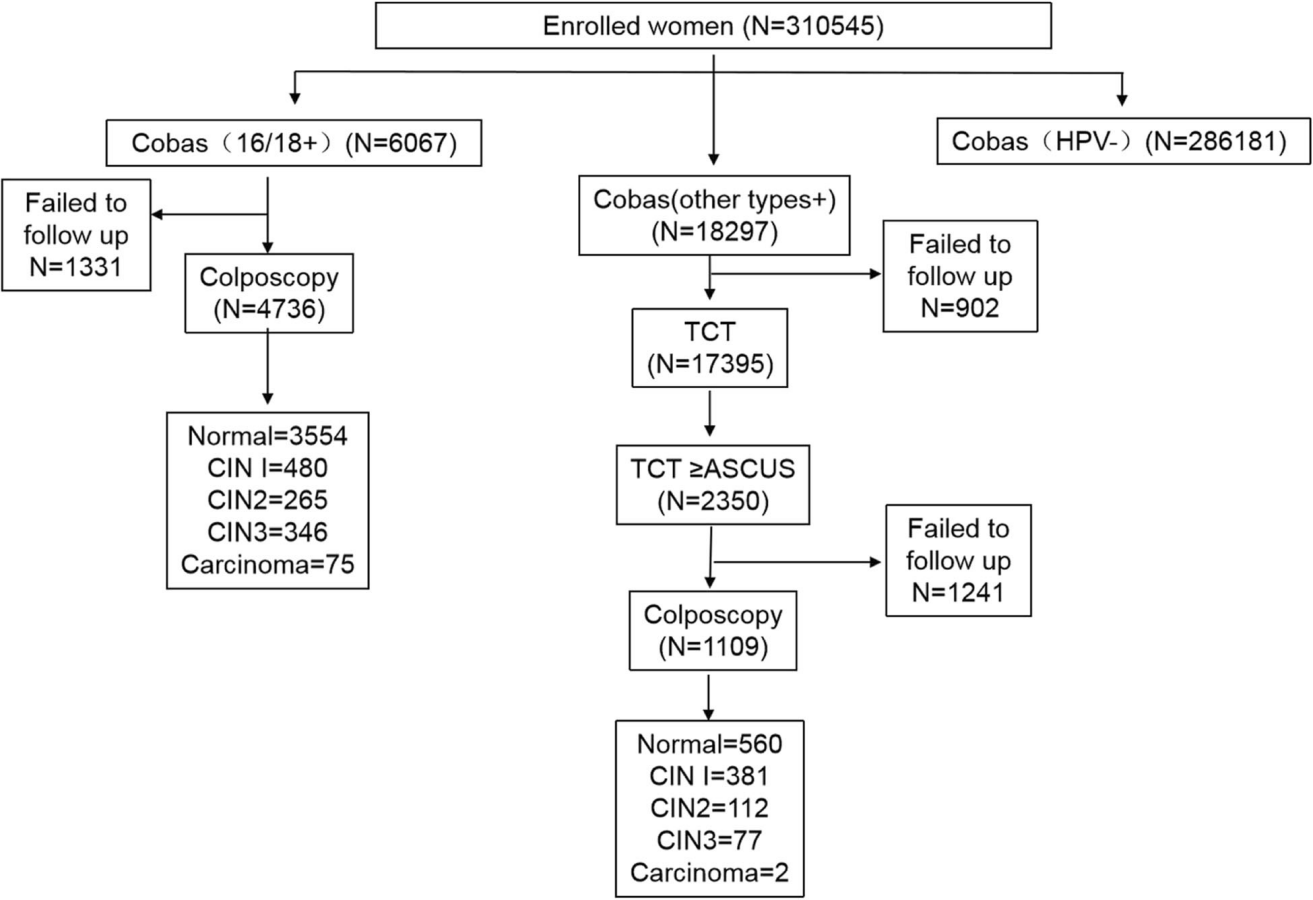
Flowchart of screening profile. Among 310,545 women, 6067 were tested positive for HPV 16/18, CIN1, CIN2, CIN3, ICC were detected in 480, 265, 346, 75 cases, respectively. Eighteen thousand two hundred ninety-seven were tested positive for other hr-HPV genotypes, CIN1, CIN2, CIN3, ICC were detected in 381, 112, 77, 2 cases, respectively

Three hundred seven thousand seventy-one female received complete screening process finally (1331 female with HPV 16 and HPV 18 and 1241 female with other positive hr-HPV were failed to follow up for different reasons, such as changes of the workplace, health problems or other personal reasons, 902 samples failed to prepare slides for liquid-based cytology for having too little cells.) 6067(1.95%) female were tested positive for HPV 16 and HPV 18, including HPV 16 only, HPV 16+ other high risk (OHR), HPV 18 only, HPV 18+ OHR, HPV 16 + 18, and HPV 16+ 18+ OHR types. 4736 (1.53%) among them, received colposcopy, CIN1, CIN2, CIN3, ICC were detected in 480 (0.15%) cases, 265 (0.086%) cases, 346 (0.11%) cases, 75 (0.024%) cases, respectively. 18,297 (5.89%) female were tested positive only for other hr-HPV genotypes, and undertook triage using liquid-based cytology. 2350 (0.76%) cases with the results ≥ ASCUS were referred to colposcopy directly, and CIN1, CIN2, CIN3 and ICC were detected in 381 (0.12%) cases, 112 (0.036%) cases, 77 (0.025%) cases, 2 cases, respectively (Fig. 1).

### Correlation between *Candida albicans*, Gardnerella, TV and the detection of cervical lesions stratified by different hr-HPV types

**Fig. 2:**
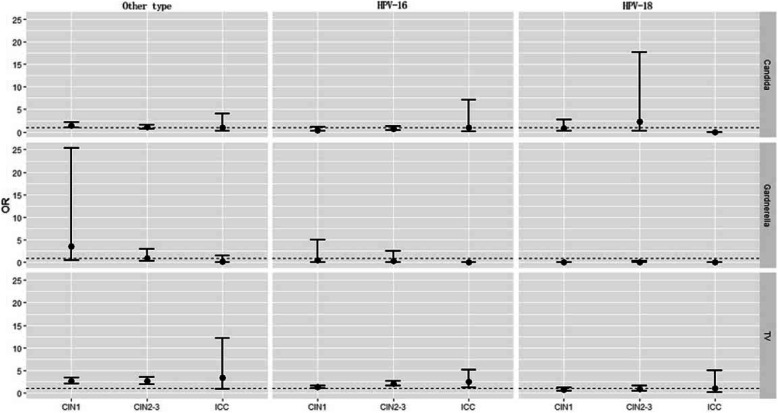
Univariate analyses of *Candida albicans*, Gardnerella, TV for the risk of CIN1/CIN2–3/ICC in different hr-HPV types

The distribution of potential risk factors among CIN/ICC and normal participants are shown in Additional file 2. Univariate analysis of the correlation between different vaginal infection (*Candida albicans*, Gardnerella, TV micro-environment) and the histological results (three categories: CIN1, CIN2–3 and ICC) among different hr-HPV types is displayed in Fig. 2 and Additional file 3. In HPV 16 infection groups, when co-infected with TV, female had a significantly higher incidence of CIN 1 (OR = 1.301, 95% CI = 1.003–1.685), CIN 2–3 (OR = 2.066, 95% CI = 1.625–2.627) and ICC (OR = 2.546, 95% CI = 1.248–5.191). In HPV 18 positive groups, the co-infection of TV didn’t increase the risk of CIN1, CIN2–3, or ICC. In all the different hr-HPV types, no significant correlation was found between *Candida albicans*, Gardnerella co-infection, and any cervical lesions.

### Multivariate analysis of the risk of CIN/ICC by TV stratified by hr-HPV infection status

Among the overall participants, 5683 (1.83%) participants were infected with TV; among them, 1952 (0.63%) female were co-infected with hr-HPV. Among the co-infection population, 1643 cases had normal cytology or histologic diagnosis, 171 cases had CIN 1, 60 cases had CIN 2, 67 cases had CIN3 and 11 cases had ICC (Table 1).

**Table 1:** Histologic diagnosis distribution in 1952 h-HPV and TV co-infection population

HPV genotype		Normal^a^ n (%)^b^	CIN1 n (%)	CIN2 n (%)	CIN3 n (%)	Cancer n (%)	Total n
16	Single-infection	1127(61.48)	234(12.77)	161(8.78)	260(14.18)	51(2.78)	1833
	Co-infection	35(71.69)	68(13.85)	23(4.68)	42(8.55)	6(1.22)	491
16 + OHR	Single-infection	492(63.48)	121(15.61)	75 (9.68)	75 (9.68)	12(1.55)	775
	Co-infection	132(69.84)	25(13.23)	15(7.94)	15(7.94)	2(1.06)	189
16 + 18	Single-infection	24(55.81)	10(23.26)	5(11.63)	4(9.3)	0(0)	43
	Co-infection	5(45.45)	3(27.27)	1(9.09)	1(9.09)	1(9.09)	11
16 + 18 + OHR	Single-infection	30(63.83)	6(12.77)	9(19.15)	1(2.13)	1(2.13)	47
	Co-infection	3(50)	1(16.67)	1(16.67)	1(16.67)	0(0)	6
18	Single-infection	401(77.12)	82(15.77)	16(3.08)	10(1.92)	11(2.12)	520
	Co-infection	87(82.86)	13(12.38)	3(2.86)	1(0.95)	1(0.95)	105
18 + OHR	Single-infection	169(73.48)	51(22.17)	6(2.6)	4(1.74)	0(0)	230
	Co-infection	15(46.87)	14(43.75)	2(6.25)	1(3.13)	0(0)	32
OHR	Single-infection	16,916(96.95)	357(2.05)	105(0.60)	69(0.39)	2(0.01)	17,449
	Co-infection	1049(93.83)	47(4.20)	15(1.34)	6(0.54)	1(0.09)	1118

^a^: female who have normal histologic or cytology results. OHR: other high risk type. (^b^percentages are calculated in turn; OHR-other high risk)

Multivariate logistic regression analyses were used for analyzing the risk of CIN 1, CIN 2–3 and ICC by TV stratified by different hr-HPV infection status. After adjusting the age, education, number of pregnancies, number of births, menopause, *Candida albicans*, Gardnerella and miscellaneous bacteria density, method of contraception, lifetime sex partners and poverty, it was found that among total female infected with hr-HPV, TV increased the risk of CIN1 (OR 1.18, 95% CI: 1.42–2.31), but it did not increase the risk of CIN2–3 and ICC. For different HPV types, TV increased the risk of CIN2–3 among the female infected with HPV16 (single HPV16 or simultaneous infection of HPV 16 and other high-risk HPV types) (OR 1.71, 95% CI: 1.16–2.53). For there were only 77 ICC female in this study, no efficient results were found among HPV 16 positive ICC female. No positive correlation were found between TV and CIN 1/CIN 2–3/ICC among none HPV16 positive female in this study (Table 2).

**Table 2:** Multivariate logistic regression analyses of the risk of CIN1/CIN2–3/ICC by TV stratified by hr-HPV infection status

	CIN1			CIN2–3			ICC		
	n	OR	*p*	n	OR	*p*	n	OR	*p*
Total	861	1.18(1.42–2.31)	<.0001	800	1.17(0.88–1.55)	0.2735	77	0.99(0.36–2.75)	0.9898
16	371	0.69(0.48–1.01)	0.0501	590	1.71(1.16–2.53)	0.0069	64	–	–
18	149	1.96(0.85–4.51)	0.112	55	2.18(0.75–6.34)	0.1544	12	–	–
OHR	535	0.73(0.49–1.08)	0.1147	344	0.90(0.56–1.45)	0.6704	15	0.36(0.12–1.06)	0.0633

Adjusted for age, the highest level of education, number of pregnancies, number of births, menopause, miscellaneous bacteria density, poverty, method of contraception, lifetime sex partners, gardnerella and candida spp. (OHR-other high risk)

### Potential risk factors of hr-HPV mono-infection or co-infection with TV

**Fig. 3:**
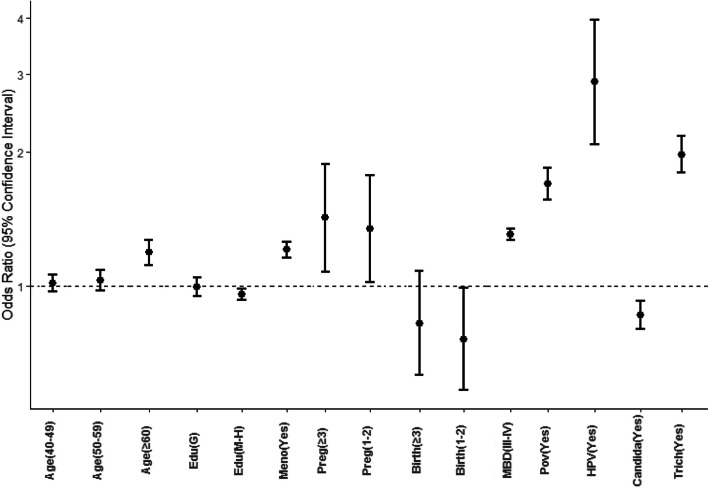
Multivariate analysis of hr-HPV infection. Age, highest level of education, number of pregnancies and number of births were set as rank variables. * MBD means miscellaneous bacteria density

To find the risk factors of hr-HPV infection, multivariate logistic regression analyses were utilized. The results are shown in Fig. 3; it was found that age ≥ 60 (*p* < 0.001), number of pregnancies ≥1 (*p* < 0.001), postmenopause (*p* < 0.01), poverty (*p* < 0.01), personal HPV history (*p* < 0.01), miscellaneous bacteria density (*p* < 0.0001), trichomonas (*p* < 0.0001), were positively associated with hr-HPV infection significantly; Education(*p* = 0.0154), was not associated with hr-HPV infection; Number of birth, and candida infection were negatively correlated with hr-HPV infection (*p* < 0.0001).

Baseline features of the participants with hr-HPV and TV co-infection are shown in Table 3, primary education, intrauterine contraceptive device as a method of contraception and female with multiple sex partners were more likely to be infected with both TV and hr-HPV.

**Table 3:** Baseline features of the participants with hr-HPV and TV co-infection

	OR(95%CI)	*P*
**Education**		<.0001
Primary	ref	
Middle and high	0.424(0.377–0.476)	
Graduate	0.540(0.458–0.636)	
**Marital status**		0.1745
Married	0.729(0.462–1.150)	
Divorced or widowed	ref	
**Method of contraception**		<.0001
Non-contraception	0.236(0.207–0.270)	
Contraceptive	0.335(0.280–0.401)	
Condom	0.244(0.208–0.286)	
Intrauterine contraceptive device	ref	
**Lifetime sex partners**		<.0001
1	0.009(0.008–0.010)	
≥ 2	ref	

## Discussion

Though it is recommended to begin primary hrHPV screening at age 25 years in the interim clinical guidance of ASCCP in 2015 [18], it raises a number of concerns and questions. When compared to the same strategy starting at 30 years of age, starting at 25 years of age resulted in much higher detection of CIN3+. Still progression to cancer is uncommon, so It is unclear that the identification of these women with CIN3+ would translate into a meaningful reduction of cervical cancer [23]. Furthermore, the panel had concerns regarding the potential harms of beginning primary hrHPV screening at age 25 years, particularly with regard to the number of colposcopies, despite the increased detection of disease [24]. According to the report “Global Cancer Statistics 2018”, cervical cancer ranks the fourth in the incidence and mortality of female tumors worldwide, and the second in the rate of female tumors in developing countries [1]. HPV infection is a necessary but insufficient condition for cervical cancer [25]; the number of cervical cancer patients is far lower than the number of HPV infection female [26]. With the development of microbiome, more studies have shown that the composition and changes of the cervical-vaginal microenvironment are closely related to HPV invasion, persistent infection, and the occurrence and development of cervical cancer [27, 28].

In our study, the prevalence of hr-HPV in this region was lower than the hr-HPV prevalence from other parts of China (from 9.03 to 16.8%) [17]. The cervical cancer vaccine had not been introduced in China at the time of screening, so all the female had not been vaccinated against HPV yet. This is the first time we have relatively accurate data on hr-HPV infection rates in central China.

It was found that Candida spp. was not associated with the detection of cervical lesions in this study, consistent with previous studies [5, 13, 14]. Gardnerella vaginalis was not the risk factor of CIN or ICC in our research, either. Gardnerella vaginalis was considered to possess characteristics which are essential for the pathogenesis of BV [6, 29–31], including the production of sialidase [31] and vaginolysin [30, 32]., Although some research found that BV played a functional role in female with CIN or ICC [2, 3], the correlation between Gardnerella and the progression of cervical lesions had yet to be described. In this study, Gardnerella has had a low prevalence and was only detected in 1050 (0.34%) female, and co-infected with Gardnerella didn’t increase the risk of CIN or ICC. In the vagina of female without BV, Gardnerella vaginalis was also detected, although it had a lower abundance and prevalence [33, 34]. There was enormous substantial genetic diversity within Gardnerella vaginalis [16, 35], and virulence potential differentiate significantly between various genetic types/clades [15]; this may correlate with the result in our study.

Several pieces of research had concerned the relationship between TV, HPV and CIN/ICC, but their conclusions were inconsistent. Gweneth’s study found that trichomonas vaginitis was associated with hr-HPV infection (specifically type 16) significantly, but no correlation between trichomonas and cervical lesions [10]. Ishita Ghosh’s study found that the higher risk of cervical cancer observed in the female co-infected with HPV and TV was without any enhanced risk of CIN [5]. But both of them were small-scale studies. Rui-Mei Feng’s pooled analysis found that current TV-positive female had an increased risk for hr-HPV infection compared with currently TV negative female. Both past and current TV-positive female had a decreased risk for CIN 2+ [9], but hr-HPV was detected by Hybrid Capture 2 and HPV genotype can’t be differentiated, TV was diagnosed by thinprep cytologic test in that research, and this method had a lower detection rate.

In this study, 5683 among the overall participants were TV positive and 1952 (34.35%) among these, were co-infected with hr-HPV. Co-infection with TV increased the risk of CIN 1 among female with hr-HPV, and increased the risk of CIN 2–3 among female with HPV 16. It was expected that TV was often associated with more severe inflammatory reaction and HPV persistence, and was likely closely related to the invasion, persistent infection of HPV 16. HPV including hr-HPV infections are mostly temporary. The virus can be cleared through host immune responses spontaneously, only a small number of infections persisted and progressed to cervical cancer [36]. HPV 16 was found to be the most prevalent hr-HPV type with the highest risk among all hr-HPVs to progress to CIN2–3 and ICC [37]. Studies found that the outcome of HPV infection was closely related to the local micro-environmental of the cervix [27]. A lot of research has discovered that TV led to an imbalance of the vaginal flora, which inhibited or reduced the lactobacillus substantially [5], and caused severe inflammation. Evidence were found that the continuous activation of inflammatory transcription factors can lead to cervical tissue damage, and thus improved the susceptibility and carcinogenic ability of HPVl6 [10, 38]. Meanwhile, the severity of cervical lesions was correlated with the abundance and diversity of cervicovaginal flora positively and correlated with the number of lactobacilli negatively [27, 39]. Unlike CIN 1, CIN 2–3 had been considered no longer reversible and required a more definite clinical treatment, the composition and characteristics of the cervical and vaginal flora were very different [40] [2, 3].. There may be some complex host immune response to TV and HPV 16 co-infection, but the hypothesis needed further research for validation. There were only 72 ICC in this study, so the correlation between TV, HPV and ICC were inconclusive.

Both TV and genital tract HPV infection are sexually transmitted disease (STD). In the districts with lower economic levels, many people went out to the developed areas to work and couples usually work in different places, and sex act extramarital is common [17]. Risk factors including primary education, intrauterine contraceptive device as a method of contraception and female with multiple sex partners significantly increased the odds of hr-HPV and TV co-infection. Compared with female with only one sexual partner, it was found that female with two or more lifetime sex partners had a higher risk of HPV and TV co-infection. Meanwhile, it was found that female who chose intrauterine contraceptive device as a method of contraception had a higher risk of co-infection, as compared with female who chose contraceptive or condom. Female who had primary education also were found to have a higher risk of co-infection compared with higher educated female. As TV is a STD, and it was found that two or more lifetime sex partners and choosing intrauterine contraceptive device instead of condom were associated with co-infection, it was expected that the co-infection is likely closely related to the risky sex behaviors. Sexual interactions with multiple hosts were expected to contribute to the co-infection, because each host may be infected with one kind of special pathogen species [41, 42]. The transmission and clinical progression of sexually transmitted infectious diseases may be changed by sexual interactions [43, 44]. Risk factors, including the number of pregnancies ≥1, post-menopause, poverty, personal HPV history and miscellaneous bacteria density were associated with HPV infection significantly, but they were not related to co-infection significantly.

As it was known that, this research had the largest number of female who received COBAS human papillomavirus primary testing for cervical cancer screening up to now worldwide, and it was a multisite investigation with the most massive scale about the correlation between cervical hr-HPV infection, CIN/ICC and vaginal microenvironment. The detection methods of hr-HPV and vaginal microenvironment in this study were highly sensitive and recognized internationally.

Of note, there were some limitations to our study. Because our study was a cross-sectional analysis of current hr-HPV and TV infections, one of its major limitations, which is inherent to the design, is that the association found does not imply causality. It was not clear whether there was a persistent infection among these female or not, so follow-up of TV-infected women to assess whether new CIN detection or CIN stage progression occurs would result in a much more clarifying study to support our findings. Besides, there are some participants who failed to be followed up for a variety of reasons in the screening process. Chlamydia has been detected less and less, so it was not included in this study. HIV-infection status and smoking habit has not been included in this study.

## Conclusions

From this large population-based study, it was found that Positive TV was correlated with hr-HPV infection and co-infection with TV increased the risk of CIN 1 among female infected with hr-HPV, co-infection with TV increased the risk of CIN 2–3 among female infected with HPV 16. From what has been discussed above, co-infection of TV and HPV 16 is a significant risk factor for the detection of cervical lesions. For our study was a cross-sectional analysis, there were some limitations, and follow-up of TV-infected women to assess whether new CIN detection or CIN stage progression occurs will be the next step of our work.

## Supplementary information

**Additional file 1.** . The content of the questionnaire.

**Additional file 2..** Distribution of potential risk factors among CIN/ICC and normal participants.

**Additional file 3. **Correlation between *Candida albicans*, Gardnerella, TV and the progression of cervical lesions stratified by different hr-HPV types

## Data Availability

The datasets used and/or analysed during the current study are available from the corresponding author on reasonable request.

## Abbreviations

hr-HPV: High risk HPV
CIN: Cervical intraepithelial neoplasia
ICC: Invasive cervical cancer
TV: Trichomonas vaginitis
BV: Bacterial vaginitis
MBD: Miscellaneous bacteria density
NILM: Negative for intraepithelial lesion or malignancy
AGC: Atypical glandular cells
SIL: Squamous intraepithelial lesion
LSIL: Low grade squamous intraepithelial lesion
HSIL: High grade squamous intraepithelial lesion
ASC-US: Atypical squamous cells of undetermined significance
ASC-H: Atypical squamous cells not possible exclude HSIL
SCC: Squamous cell carcinoma
ECC: Endocervical curettage
CIs: Confidence intervals
OHR: Other high risk
STD: Sexually transmitted disease
