# Supplementary material for: Co-infection with trichomonas vaginalis increases the risk of cervical intraepithelial neoplasia grade 2–3 among HPV16 positive female: a large population-based study
Source: BMC Infect Dis. 2020 Sep 1;20:642. doi: 10.1186/s12879-020-05349-0 (PMC7466445; doi:10.1186/s12879-020-05349-0)
Supplement: Supplementary file 1 — Additional file 1. . The content of the questionnaire. [file 12879_2020_5349_MOESM1_ESM.doc]

**Additional files**

Additional file 1. The content of the questionnaire

| age |  |
| --- | --- |
| marital status |  |
| ethnicity |  |
| the highest level of education |  |
| whether in menopause |  |
| date of last menstruation |  |
| history of past HPV infection |  |
| family history of cancer |  |
| number of pregnancies |  |
| number of births |  |
| method of contraception |  |
| number of lifetime sex partners |  |
| whether being a poverty alleviation target |  |
