# Supplementary material for: Co-infection with trichomonas vaginalis increases the risk of cervical intraepithelial neoplasia grade 2–3 among HPV16 positive female: a large population-based study
Source: BMC Infect Dis. 2020 Sep 1;20:642. doi: 10.1186/s12879-020-05349-0 (PMC7466445; doi:10.1186/s12879-020-05349-0)
Supplement: Supplementary file 2 — Additional file 2.. Distribution of potential risk factors among CIN/ICC and normal participants. [file 12879_2020_5349_MOESM2_ESM.docx]

Additional file 2. Distribution of potential risk factors among CIN/ICC and normal participants.

| **Characteristics** | **Normal^#^** | **CIN/ICC** |
| --- | --- | --- |
|  | **n** | **n** |
| Women screened | 305333 | 1738 |
| **Age (years)** |  |  |
| 30-39 | 52822 | 255 |
| 40-49 | 108219 | 633 |
| 50-59 | 112169 | 643 |
| ≥60 | 32123 | 207 |
| **Education** |  |  |
| Primary | 143501 | 788 |
| Middle and high | 129835 | 794 |
| Graduate | 31997 | 156 |
| **Marital status*** |  |  |
| Married | 270737 | 1229 |
| Divorced or widowed | 1918 | 25 |
| **Menopause** |  |  |
| No | 173905 | 972 |
| Yes | 131428 | 766 |
| **No. pregnancies*** |  |  |
| 0 | 11900 | 32 |
| 1-2 | 152488 | 787 |
| >=3 | 139950 | 919 |
| **No. live births*** |  |  |
| 0 | 12511 | 39 |
| 1--2 | 254324 | 1439 |
| ≥3 | 37419 | 260 |
| **MBD*** |  |  |
| Ⅰ/Ⅱ | 279223 | 1051 |
| Ⅲ/Ⅳ | 21394 | 687 |
| **Gardnerella*** |  |  |
| No | 299592 | 1713 |
| Yes | 1025 | 25 |
| **Candida spp*** |  |  |
| No | 286962 | 1630 |
| Yes | 13655 | 108 |
| **Trichomonas*** |  |  |
| No | 295243 | 1429 |
| Yes | 5374 | 309 |
| **Method of contraception*** |  |  |
| Non-contraception | 162854 | 612 |
| Contraceptive | 47332 | 558 |
| Condom | 25360 | 193 |
| Intrauterine contraceptive device | 37420 | 264 |
| **Lifetime sex partners*** |  |  |
| 1 | 267782 | 518 |
| ≥2 | 676 | 126 |
| **Object for poverty alleviation*** |  |  |
| No | 299105 | 1714 |
| Yes | 5233 | 24 |
| **Personal HPV History*** |  |  |
| No | 269638 | 1600 |
| Yes | 23696 | 102 |

#: female who tested negative for hr-HPV or have normal histologic results. ***:** there were some missing values
