# Supplementary material for: Co-infection with trichomonas vaginalis increases the risk of cervical intraepithelial neoplasia grade 2–3 among HPV16 positive female: a large population-based study
Source: BMC Infect Dis. 2020 Sep 1;20:642. doi: 10.1186/s12879-020-05349-0 (PMC7466445; doi:10.1186/s12879-020-05349-0)
Supplement: Supplementary file 3 — Additional file 3. Correlation between Candida albicans, Gardnerella, TV and the progression of cervical lesions stratified by different hr-HPV types [file 12879_2020_5349_MOESM3_ESM.docx]

Additional file 3. Correlation between Candida Albicans, Gardnerella, TV and the progression of cervical lesions stratified by different hr-HPV types

|  |  |  | OR(95%CI) |  |
| --- | --- | --- | --- | --- |
|  |  | CIN1 | CIN2-3 | ICC |
| HPV16 | TV | 1.301(1.003,1.685) | 2.066(1.625-2.627) | 2.546(1.248-5.191) |
|  | Gardnerella | 0.462(0.042-5.105) | 0.35(0.049-2.488) | 0 |
|  | Candida | 0.449(0.256-1.089) | 0.734(0.415-1.296) | 0.954(0.128-7.124) |
| HPV18 | TV | 0.815(0.522-1.272) | 0.856(0.428-1.71) | 1.07(0.231-4.952) |
|  | Gardnerella | 0 | 0.083(0.02-0.337) | 0 |
|  | Candida | 0.852(0.273-2.655) | 2.322(0.3-17.69) | 0 |
| OHR | TV | 2.658(2.097-3.368) | 2.661(1.987-3.564) | 3.422(0.964-12.141) |
|  | Gardnerella | 3.51(0.486-25.32) | 0.952(0.298-3.038) | 0.217(0.03-1.593) |
|  | Candida | 1.416(0.92-2.181) | 1.051(0.693-1.593) | 0.969(0.235-3.991) |
